# On Neuroma and Neuro-Fibromatosis

**Published:** 1901-03

**Authors:** 


**On Neuroma and Neuro-Fibromatosis.** By Alexis Thomson. Pp. viii., 168. Edinburgh: Turnbull & Spears. 1900.

This is a very good and interesting monograph, which will no doubt be in future often referred to as an authority on the subject of which it treats. The author's opportunities of having personally observed the large number—for these rare growths—of fifteen cases have given him a practical acquaintance with them. He has given a new classification of neuromata and allied conditions, which is founded on an anatomical basis. Each variety is described, and there is appended a further summary of the most important cases from the literature of the subject, and of his own cases. Dr. Alexis Thomson points out that the true neuromata—the rarest of all forms—are connected with the sympathetic nervous system, except in one case, which is open to doubt. He separates, on good practical grounds, the solitary or circumscribed tumours of nerves from the general disease, neuro-fibromatosis, and includes under the former the painful subcutaneous tubercle of Wood. Under the title of "Diffuse or Generalised Neuro-fibromatosis" fall von Recklinghausen's disease and elephantiasis nervorum (Bruns). In neuro-fibromatosis the chief seat of the pathological process is the endoneural connective tissue between the individual nerve fibres. With regard to the effects of fibromatosis on the nerve-fibres and the disputed question as to whether it causes degeneration of fibres, the author believes that such degeneration may occur in cases of generalised neuro-fibromatosis, but is exceptional, and of comparatively little importance. Some good examples are given of that most interesting form, the plexiform neuro-fibroma. Towards the end of the book there is a useful general summary with two tables of published cases from all sources, and the work concludes with a bibliography. This renders the book, with the original observations, a very complete study of the subject. It is illustrated with a number of beautiful plates, some of patients and some of pathological specimens, both macroscopical and microscopical. These plates much enhance the value of the text, which is lucidly written, and the clearness of the descriptions is largely responsible for a very interesting monograph, on which the author is much to be congratulated. The publisher has also done his work well.

